# Chromatin target of protein arginine methyltransferase regulates invasion, chemoresistance, and stemness in epithelial ovarian cancer

**DOI:** 10.1042/BSR20190016

**Published:** 2019-04-16

**Authors:** Xiaojie Feng, Lei Li, Li Wang, Suxia Luo, Xupeng Bai

**Affiliations:** 1 Department of Gynecologic Oncology, The Affiliated Cancer Hospital of Zhengzhou University, Zhengzhou, Henan, China; 2 Cancer Care Centre, St George Hospital, Kogarah, New South Wales, Australia; 3 St George and Sutherland Clinical School, Faculty of Medicine, UNSW Sydney, New South Wales, Australia

**Keywords:** CHTOP, cisplatin, metastasis, ovarian cancer, prognosis, stemness

## Abstract

Ovarian cancer is one of the most common gynecological cancers with a high mortality rate in females. Chromatin target of protein arginine methyltransferase (CHTOP) is an important intracellular protein that regulates the transcriptional activation of several oncogenic genes in glioblastomagenesis and controls mature mRNA export as a component of TRanscription-Export complex. However, the role of CHTOP in ovarian cancer is unclear. In the present study, we investigated the correlation between tumor-derived CHTOP expression and prognosis and explored its role in the malignant behaviors of epithelial ovarian cancer cells. We found that higher expression of CHTOP was associated with a lower disease-free survival (DFS) rate in ovarian cancer patients. Also, CHTOP was highly expressed in human ovarian cancer tissues compared with normal and adjacent tissues. Moreover, compared with IGROV-1 cell line, higher expression of CHTOP was also confirmed in the malignant ovarian cancer cell lines (OV-90 and SK-OV-3). Further results from wound-healing and Matrigel assay showed that CHTOP knockdown significantly reduced the migration and invasion ability of OV-90 and SK-OV-3 cells, while colony formation assay and apoptosis detection showed that CHTOP knockdown markedly sensitized OV-90 and SK-OV-3 cells to cisplatin treatment by inducing apoptosis. Additionally, CHTOP silence also remarkably weakened the stemness of OV-90 and SK-OV-3 through inhibiting the protein expressions of several transcriptional or surface markers of cancer stem cells. These findings first suggest that CHTOP, as a highly expressed protein in ovarian cancer, is closely associated with the malignant phenotypes of epithelial ovarian cancer cells, including metastasis, chemoresistance, and stemness, which highlights a promising role of CHTOP in ovarian cancer targeted therapy.

## Introduction

Ovarian cancer is one of the most common gynecological cancers with a high mortality rate in females [1]. The average 5-year survival rate for ovarian cancer was only 46% [2]. Thereinto, epithelial ovarian cancer accounts for approximately 85–95% of ovarian cancer cases, and over 70% of epithelial ovarian cancer cases are diagnosed at an advanced stage due to the poor screen techniques in early development stages [3]. Chemotherapy is an essential therapeutic option in treating ovarian cancer. Unfortunately, current chemotherapeutic regime is ineffective after several administration courses [4]. The recurrence risk after regular chemotherapy was approximately 80–85% in advanced epithelial ovarian cancer patients [5]. Thus, finding novel targeted therapeutics in combination with conventional therapy to improve the present treatments and prognosis is in urgent need.

Chromatin target of protein arginine methyltransferase (PRMT) (CHTOP) is a protein that tightly associates with facultative heterochromatin in vertebrate interphase cells. PRMT-1 and PRMT-5 can regulate CHTOP activity by competing for binding to it and methylating the arginines of CHTOP in asymmetric and symmetric ways, respectively, which triggers opposite effects on CHTOP. For example, CHTOP and PRMT-1 are involved in the transcriptional activation of estrogen receptor α (ERα)-targeted genes by inducing E2-dependent recruitment of ERα to the promoter, whereas CHTOP and PRMT5 contribute to the down-regulation of fetal γ-globin during the developmental transition of fetal to adult hemoglobin [6,7]. In addition, CHTOP also plays an important role in mature mRNA export as a component of the TRanscription–EXport (TREX) complex through its ability to bind to the pre-mRNA splicing factor UAP56 [7,8].

The role of CHTOP in cancer is not well determined yet. A recent study observed that CHTOP was up-regulated during glioblastomagenesis [9]. Further study indicated that CHTOP can bind to the 5-hydroxymethylcytosine (5hmC)-containing promoter and recruit the methylosome, including PRMT1, PRMT5, methylosome protein 50 (MEP50), and enhancer of rudimentary homolog (ERH), to chromatin in a ten-eleven translocation methylcytosine dioxygenase 1 (TET1)-dependent manner, facilitating PRMT-1-mediated methylation of arginine 3 of histone H4 (H4R3) and promoting the transcriptional activation of key genes involved in glioblastomagenesis, such as epidermal growth factor receptor (EGFR), AKT3, cell division protein kinase 6 (CDK6), and B-Raf (BRAF) [9]. This study sheds light on the role of CHTOP in cancer and makes it an attracted target for cancer treatment. However, the role of CHTOP in epithelial ovarian cancer is not clear yet.

In the present study, we investigated the association of CHTOP expression with clinical outcomes and explored its role in the malignant behaviors of epithelial ovarian cancer cells. Our data indicate that CHTOP overexpression is associated with a poor prognosis in ovarian cancer patients and an enhanced phenotype of metastasis, chemoresistance, and stemness in epithelial ovarian cancer cells, highlighting the potential of CHTOP as a novel therapeutic target in dealing with ovarian cancer.

## Materials and methods

### Cell culture

Epithelial ovarian cancer cell lines, including IGROV-1, SK-OV-3, and OV-90, were purchased from American Type Culture Collection (ATCC, Rockville, U.S.A.). Ovarian surface epithelial cell line (HOSE) was purchased from Cell Bank of Type Culture Collection of Chinese Academy of Sciences (Shanghai, China). SK-OV-3 cells were cultured in McCoy’s 5A Medium (HyClone, U.S.A.) supplemented with 10% fetal bovine serum (FBS, Gibco, U.S.A.) and 1× antibiotic–antimycotic solution (Gibco, U.S.A.). OV-90 cells were cultured in a 1:1 mixture of MCDB 105 medium (HyClone, U.S.A.) and Medium 199 (HyClone, U.S.A.) supplemented with 15% FBS and 1× antibiotic–antimycotic solution. HOSE cells were cultured in RPMI-1640 medium supplemented with 10% FBS and 1× antibiotic–antimycotic solution. All cell lines were incubated at 37°C with 5% CO_2_ in a humidified incubator.

### Western blot analysis

Total protein was extracted using radioimmunoprecipitation (RIPA) lysis buffer (Thermo Scientific, U.S.A.) and quantitated by BCA protein assay (Thermo Scientific, U.S.A.). Protein samples were separated by 10– 12% sodium dodecyl sulfate/polyacrylamide gel electrophoresis (SDS/PAGE) and then transferred to 0.45 μm polyvinylidene fluoride (PVDF) membrane (Millipore, U.S.A.). Following blocking with 5% non-fat milk (BD Bioscience, U.S.A.), membranes were incubated with primary antibodies overnight at 4°C and then incubated with HRP–conjugated secondary antibodies for 1 h at room temperature. Finally, protein bands were detected using enhanced chemiluminescence (ECL) detection kit (Pierce Chemical, U.S.A.) and the ImageQuant LAS4000 system (GE Healthcare, U.S.A.). GAPDH was used as the loading control. The antibodies used in the present study are listed in Table 1.

**Table 1:** Antibodies used for western blot and immunofluorescence

Antibodies	Brand	Species	Type	Dilution
Rabbit anti-C1ORF77 (CHTOP)	Abcam	Human	Monoclonal	1:500 (WB)
				1:100 (IF)
Rabbit anti-uncleaved PARP-1	Abcam	Human	Monoclonal	1:1000
Rabbit anti-cleaved PARP-1	Abcam	Human	Monoclonal	1:1000
Rabbit anti-active caspase-3	Abcam	Human	Monoclonal	1:1000
Rabbit anti-Bax	Abcam	Human	Monoclonal	1:1000
Rabbit anti-Bcl-2	Abcam	Human	Monoclonal	1:1000
Rabbit anti-Bcl-xL	Abcam	Human	Monoclonal	1:1000
Rabbit anti-CD44	Abcam	Human	Monoclonal	1:1000
Rabbit anti-OCT-4	Abcam	Human	Monoclonal	1:1000
Rabbit anti-SOX-2	Abcam	Human	Monoclonal	1:1000
Rabbit anti-NANOG	Abcam	Human	Monoclonal	1:1000
Rabbit anti-GAPDH	Abcam	Human	Monoclonal	1:5000
Rabbit anti-ALDH1	Abcam	Human	Monoclonal	1:1000
Goat anti-rabbit IgG-HRP	CST	Human	IgG	1:2000
Goat anti-rabbit Alexa Fluor® 488 Dye	Invitrogen	Human	IgG	1:1000 (IF)

Abbreviations: IF, immunoflourescence; WB, western blot; PARP-1, poly (ADP-ribose) polymerase 1; Bcl-2, B-cell lymphoma 2; Bax, Bcl-2-associated X protein; Bcl-xL, B-cell lymphoma-extra large; OCT-4, octamer-binding transcription factor 4; SOX-2, SRY (sex determining region Y)-box 2; ALDH1, aldehyde dehydrogenase 1.

### CHTOP-specific siRNA transfection

CHTOP-specific siRNA (si-CHTOP) and negative control siRNA (si-con) were synthesized by RiboBio Technologies (Guangzhou, China). SiRNA transfection was performed using Lipofectamine RNAiMAX transfection reagent (Invitrogen, U.S.A.). Briefly, cells seeded in 75 cm^2^ flask were transfected with siRNAs and incubated for 48 h before following experiments according to manufacturer’s specifications.

### Cell proliferation assay

After CHTOP knockdown, cells were seeded in the 96-well plate at a density of 1000 cells per well and cultured for 7 consecutive days. The cell proliferation rate was detected by CyQUANT® Cell Proliferation Assay Kit (Life Technologies, U.S.A.) according to manufacturer’s protocol. The fluorescence was measured using a Flex Station 3 Multifunctional Microplate Reader (Molecular Devices, U.S.A.) (excitation: 485 nm; emission: 530 nm).

### Immunofluorescence

Cells seeded on glass coverslips were fixed using methanol and then blocked with 2% bovine serum albumin (BSA, Sigma–Aldrich, U.S.A.) for 1 h at room temperature. Then they were incubated with primary antibodies overnight at 4°C and Alexa Fluor™ 488 antibody for 1 h at room temperature. DAPI (Thermo Scientific, U.S.A.) was used for nucleus staining. Immunofluorescence was photographed immediately using an inverse fluorescence microscope (Olympus, Japan). The antibodies used in the present study are listed in Table 1. The mean intensity of fluorescence from five randomly selected fields was scored as negative (−), weak (+), moderate (++), and strong (+++).

### Matrigel invasion assay

A total of 2 × 10^4^ cells were seeded in a Matrigel Transwell® insert chamber (Corning, U.S.A.) with 500 µl of serum-free RPMI-1640 medium (HyClone, U.S.A.), while 750 µl complete medium was added to the bottom of each well. Cells were incubated for 48 h and then stained with Differential Quik Stain Kit (Allegiance Healthcare, U.S.A.). The number of cells that invaded through Matrigel or control chamber was counted in five randomly selected fields by a light microscope (Olympus, Japan). Invasion ability = [(mean number of cells invading through Matrigel chamber)/(mean cells migrating through control chamber)] × 100%.

### Wound-healing assay

Monolayer cells seeded in a six-well plate at the density of 3 × 10^5^ per well were scraped by a sterile 200-µl pipette tip, leading to a clear straight wound. Then cells were washed using 1× PBS and then cultured in the medium for 48 h. Representative images were obtained at 0 and 48 h using a light-field microscope at 40× magnification.

### Drug preparation

Cisplatin was purchased from Sigma–Aldrich (U.S.A.). The store solution of cisplatin was prepared in dimethyl sulfoxide (DMSO, Sigma–Aldrich, U.S.A.), while the working solution of cisplatin was prepared in cell culture medium using store solution and the final concentration of DMSO was 0.1%. The concentration of cisplatin used in the present study is half of IC_50_ of the corresponding cell line; 0.1% DMSO was used as the control treatment.

### Cell apoptosis analysis

Cells treated with siRNA for 72 h and/or cisplatin for 24 h were collected and incubated with Annexin V-FITC (Thermo Scientific, U.S.A.) for 15 min at room temperature in the dark. Cell early apoptosis was then analyzed in FACSCalibur™ platform (BD Biosciences).

DNA breaks (late apoptosis) were detected by the *In Situ* Cell Death Detection Kits (Roche Applied Science, U.S.A.). In brief, fixed cells were incubated with 20 μg/ml protease K for 15 min at room temperature and then the TUNEL reaction mixture for 1 h at 37°C in a humidified incubator. Finally, cells were incubated in turn with converter-peroxidase and the 3,3′-diaminobenzidine substrate. Images were obtained immediately using a light microscope at 200× magnification. The percentage of TUNNEL-positive cells were calculated from five randomly selected fields.

### Mammosphere-formation assay

Cells were seeded in a six-well ultra-low attachment round bottom plate (Corning, U.S.A.) at a density of 2000 per well in serum-free DMEM/F12K medium (HyClone, U.S.A.) supplemented with 1× B27 (Gibco, U.S.A.), 20  ng/ml epithermal growth factor (EGF, Sigma–Aldrich, U.S.A.), and 20  ng/ml basic fibroblast growth factor (bFGF, Sigma–Aldrich, U.S.A.). The number of spheres (diameter ≥ 50 μm) was counted after 5–7 days by an inverted phase microscope (Olympus, Japan) fitted with an ocular eyepiece. Mammosphere-formatting efficiency was calculated as: the number of spheres per 2000 cells.

### Immunohistochemistry

The present study was carried out in accordance with the recommendations of the Guide for the Use of Human Samples of Zhengzhou University with written informed consent from all subjects. All subjects gave written informed consent in accordance with the Declaration of Helsinki. The protocol was approved by the Institutional Review Board of the Cancer Hospital of Zhengzhou University (Ethics Approval No. 2018831). Paraffin-embedded tissue slides were deparaffinized and rehydrated in xylene (Sigma–Aldrich, U.S.A.) and ethanol (Sigma–Aldrich, U.S.A.), respectively, and then treated by 0.01 M sodium citrate (Sigma–Aldrich, U.S.A.) in boiling water for 20 min. Subsequently, slides were incubated with the primary CHTOP monoclonal antibody for 1 h at room temperature followed by a secondary antibody for 30 min at room temperature. Control slides were treated identically but incubated with a non-specific immunoglobulin. Finally, the slides were stained with Liquid DAB+ Substrate Chromogen System (Dako, U.S.A.) and then counterstained with Harris Hematoxylin (Thermo Fisher, U.S.A.) for nucleus staining. The slides were photographed by a light-field microscope at 200× magnification. The mean intensity of immunostaining from five randomly selected fields was scored as negative (−), weak (+), moderate (++), and strong (+++).

### Statistical analysis

Kaplan–Meier analysis of overall survival (OS) and disease-free survival (DFS) with log-rank tests was provided by Kaplan–Meier plotter (www.kmplot.com/analysis) with 2017 version database (*n*=1657 and *n*=1435, respectively) and median CHTOP expression value was set as 2652 (expression range 1445–6073). Gene expression and survival data were obtained from Gene Expression Omnibus and The Cancer Genome Atlas [10]. All assays were performed at least three times and data were presented as mean ± standard error of the mean (S.E.M.). Unpaired two-tailed Student’s *t* test was performed for two-group comparisons, while one-way ANOVA with Tukey’s post hoc test was performed for multiple group comparisons using GraphPad® Prism 7. *P*<0.05 was considered statistically significant.

## Results

### Higher expression of CHTOP was associated with a lower DFS rate and was found in malignant epithelial ovarian cancer cells

**Figure 1:**
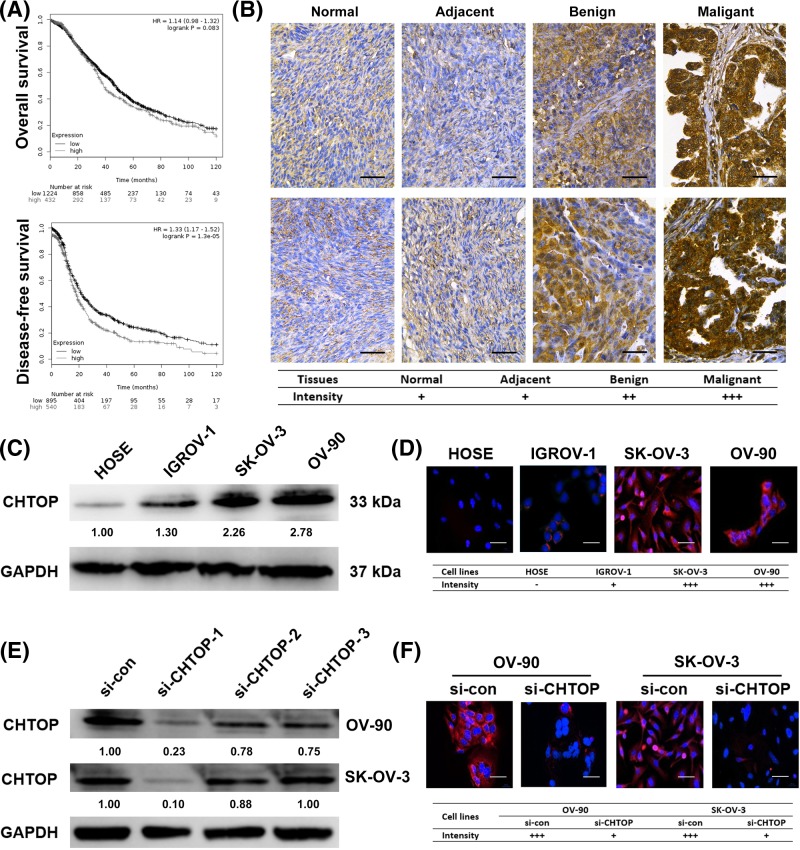
Higher expression of CHTOP was associated with a lower DFS rate and was found in malignant epithelial ovarian cancer cells (**A**) Higher expression of CHTOP was associated with a lower 10-year DFS rate in ovarian cancer patients. (**B**) CHTOP was highly expressed in human ovarian cancer tissues. Representative immunohistochemical images of normal ovarian tissues, adjacent tissues, and benign and malignant cancer tissues were obtained at 400× magnification. Brown represents CHTOP staining, while blue represents nuclear staining. (**C**) Compared with normal epithelial ovarian cell line (HOSE), higher protein expression of CHTOP was found in epithelial ovarian cancer cell lines (IGROV-1, OV-90, and SK-OV-3). The protein expression of CHTOP was tested and quantitated by immunoblot analysis and densitometric scan, respectively. GAPDH was used as the loading control. (**D**) Compared with normal epithelial ovarian cell line (HOSE), higher protein expression of CHTOP was found in epithelial ovarian cancer cell lines (IGROV-1, OV-90, and SK-OV-3). The protein expression of CHTOP was tested by immunofluorescence. Red fluorescence represents CHTOP, while blue fluorescence represents nucleus. Representative images were obtained at 200× magnification, and mean intensity of fluorescence from five randomly selected fields was evaluated. (**E**) Comparison of CHTOP knockdown efficiency among three CHTOP-targeted siRNAs. The protein expression of CHTOP was tested and quantitated by immunoblot analysis and densitometric scan, respectively. GAPDH was used as the loading control. (**F**) Effective CHTOP knockdown was confirmed by immunofluorescence. The protein expression of CHTOP was tested by immunofluorescence. Red fluorescence represents CHTOP, while blue fluorescence represents nucleus. Representative images were obtained at 200× magnification, and mean intensity of fluorescence from five randomly selected fields was evaluated.

In the present study, we first investigated the correlation of high expression of CHTOP with clinical outcomes in ovarian cancer. The results from Kaplan–Meier analysis showed that higher expression of CHTOP was associated with a significantly lower 10-year DFS rate in ovarian cancer patients (*P*<0.001) (Figure 1A). Although patients with higher expression of CHTOP in ovarian cancer tissues showed a lower 10-year OS, the difference was not significant (*P*=0.083) (Figure 1A). In addition, immunohistochemical analysis showed that CHTOP was highly expressed in benign ovarian cancer tissues as compared with normal ovarian or adjacent tissues that displayed a weak expression of CHTOP, while malignant ovarian cancer tissues showed the strongest expression of CHTOP (Figure 1B).

The data from immunoblot and immunofluorescence showed that, compared with normal epithelial ovarian cell HOSE, epithelial ovarian cancer cell lines (IGROV-1, SK-OV-3, and OV-90) were characterized by a significantly higher intracellular expression of CHTOP (Figure 1C,D). Furthermore, compared with the IGROV-1 cell line, SK-OV-3 (malignant epithelial ovarian cancer cell line), and OV-90 (metastatic epithelial ovarian cancer cell line) cell lines showed a much higher expression of CHTOP (Figure 1C,D). Thus, CHTOP was of interest in our study.

To start the study of the role of CHTOP in epithelial ovarian cancer, we first screened the si-CHTOP that can effectively silence the protein expression of CHTOP. As shown in Figure 1E, siRNA1 (sense: CAGACAGAUCCCGAAACCAAUGAUU; antisense: AAUCAUUGGUUUCGGGAUCUGUCUG) showed the most efficient knockdown effect as compared with siRNA2 and siRNA3 in both OV-90 and SK-OV-3 cells. Similar results were also confirmed by immunofluorescence (Figure 1F), which showed that the CHTOP protein expression in both OV-90 and SK-OV-3 cells can be effectively silenced by si-CHTOP. Thus, si-CHTOP-1 was selected for CHTOP knockdown in the following experiments.

### CHTOP knockdown inhibited the migration and invasion ability of epithelial ovarian cancer cells

**Figure 2:**
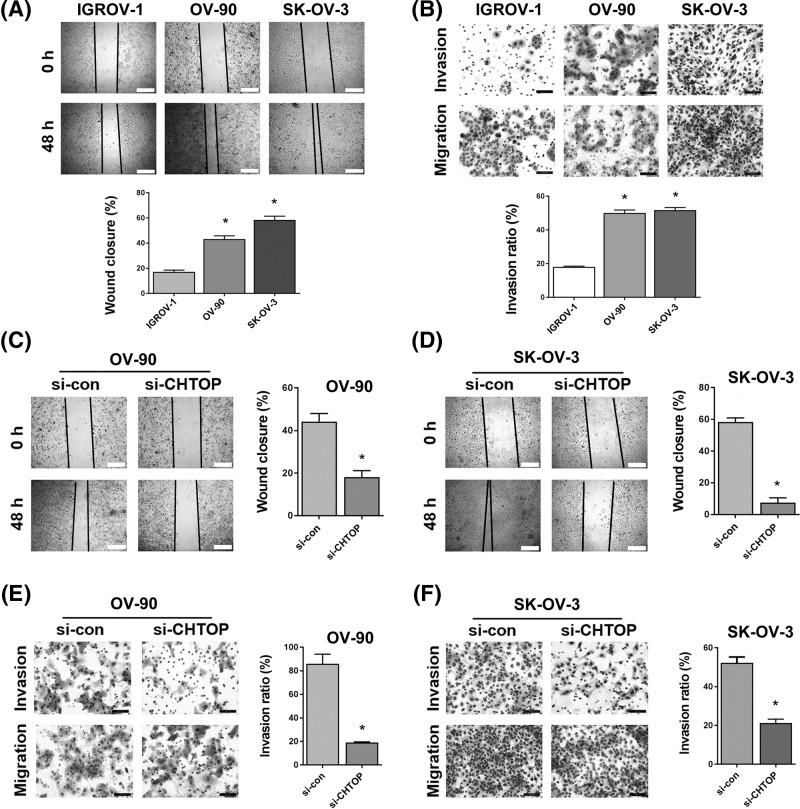
CHTOP knockdown inhibited the migration and invasion ability of epithelial ovarian cancer cells (**A**) OV-90 and SK-OV-3 cell lines showed enhanced migration capability as compared with IGROV-1 cell line. The migration potential was examined by wound-healing assay, and wound area was compared after 48 h. Representative images were obtained at 40× magnification. (**B**) OV-90 and SK-OV-3 cell lines showed enhanced invasion potential as compared with IGROV-1 cell line. The invasion potential was tested by Matrigel invasion assay. The number of cells invading through the Matrigel was counted and compared after 72 h from five randomly selective fields. Representative images were obtained at 100× magnification. (**C,D**) CHTOP knockdown significantly reduced the migration capability of OV-90 and SK-OV-3 cell lines. The migration activity was examined by wound-healing assay, and wound area was compared after 48 h. Representative images were obtained at 40× magnification. (**E,F**) CHTOP knockdown significantly reduced the invasion potential of OV-90 and SK-OV-3 cell lines. The number of cells invading through the Matrigel was counted and compared after 72 h from five randomly selective fields. Representative images were obtained at 100× magnification. Data were expressed as mean ± S.E.M. (*n*=3). *P<0.05 vs IGROV-1 or si-con group.

Since OV-90 and SK-OV-3 are characterized by a higher expression of CHTOP, we next compared the migration and invasion ability of OV-90 and SK-OV-3 with IGROV-1 in order to investigate if CHTOP is associated with the migration or invasion capability of epithelial ovarian cancer cells. As shown in Figure 2A, the results from wound-healing assay showed that, compared with IGROV-1, OV-90, and SK-OV-3 cells displayed an increased migration capability. After 48 h, more OV-90 or SK-OV-3 cells migrated into the central of the wound than IGROV-1 (Figure 2A). In addition, the results from Matrigel assay showed that, compared with IGROV-1, OV-90, and SK-OV-3 cells had much higher invasion potential (Figure 2B).

Subsequently, si-CHTOP was used to investigate the role of CHTOP in the migration and invasion capability of epithelial ovarian cancer cells. The representative images of 48-h migration activity of OV-90 and SK-OV-3 cell lines after CHTOP knockdown were shown in Figure 2C,D. The results showed that the wound-healing efficiency of OV-90 and SK-OV-3 cells was significantly weakened by CHTOP silence (Figure 2C,D). Furthermore, the invasion potential of OV-90 and SK-OV-3 cells was also markedly decreased by CHTOP knockdown (Figure 2E,F). The average invasion rates of OV-90 and SK-OV-3 cells after CHTOP knockdown were decreased to approximately 20 and 50%, respectively, compared with the corresponding si-con group (Figure 2E,F).

### CHTOP knockdown sensitized epithelial ovarian cancer cells to cisplatin treatment through inducing apoptosis

**Figure 3:**
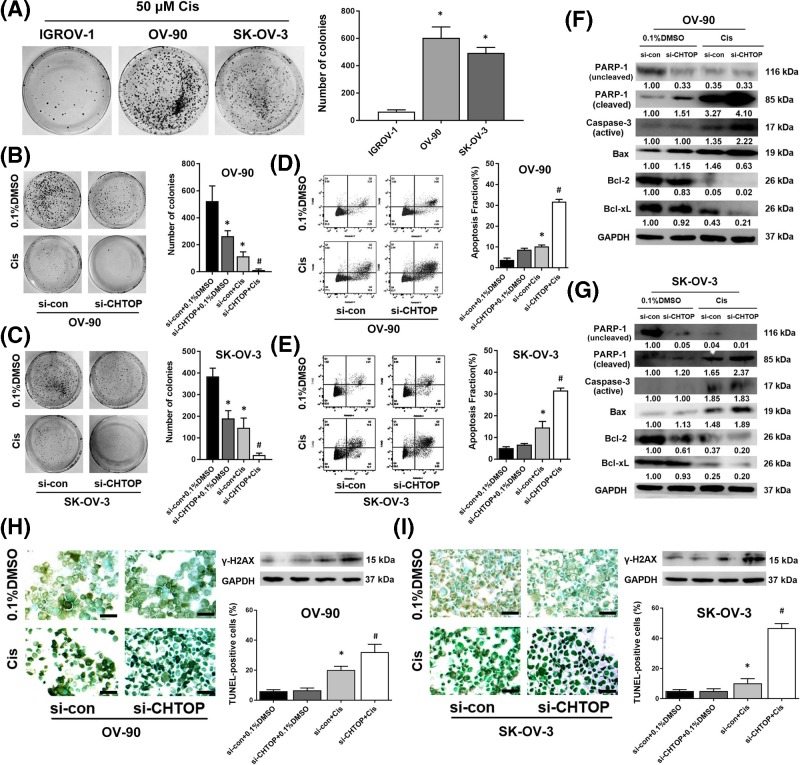
CHTOP knockdown sensitized epithelial ovarian cancer cells to cisplatin treatment through inducing apoptosis (**A**) OV-90 and SK-OV-3 cell lines showed enhanced resistance to cisplatin as compared with IGROV-1. The sensitivity of IGROV-1, OV-90, and SK-OV-3 toward cisplatin was evaluated by colony formation assay. After treatment with cisplatin (50 μM) for 24 h, the cells were cultured for colony formation. (**B,C**) Compared with cisplatin mono-treatment, combination treatment of CHTOP knockdown and cisplatin significantly reduced the colony formation ability of OV-90 and SK-OV-3 cell lines. The number of colonies was counted and compared after 14 days. (**D,E**) Compared with cisplatin mono-treatment, combination treatment of CHTOP knockdown and cisplatin significantly increased the early apoptosis fraction in both OV-90 and SK-OV-3 cell lines. Cell early apoptosis was analyzed by flow cytometry. (**F,G**) Compared with cisplatin mono-treatment, combination treatment of CHTOP knockdown and cisplatin significantly activated apoptosis-associated signaling. The protein expression was tested and quantitated by immunoblot analysis and densitometric scan, respectively. GAPDH was used as the loading control. (**H,I**) Compared with cisplatin mono-treatment, combination treatment of CHTOP knockdown and cisplatin significantly increased DNA double-strand breaks in both OV-90 and SK-OV-3 cell lines. TUNEL assay was employed to examine the DNA double-strand breaks and the percentage of TUNEL-positive cells was compared from five randomly selective fields. Representative images for TUNEL assay were obtained at 100× magnification. The expression of γH2AX was tested by immunoblot analysis, and GAPDH was employed as loading control. Data were expressed as mean ± S.E.M. (*n*=3). *P<0.05 vs IGROV-1 or si-con group, ^#^*P*<0.05 vs cisplatin-treatment group.

During preliminary experiments, we found that CHTOP knockdown can obviously inhibit the proliferation rates of OV-90 and SK-OV-3 cells (Supplementary Figure S1). Hypothesizing that CHTOP may play an important role in epithelial ovarian cancer chemoresistance, we next investigated the association of CHTOP with epithelial ovarian cancer cell chemosensitivity. The cisplatin-sensitivity of IGROV-1, OV-90, and SK-OV-3 was compared using colony formation assay. As shown in Figure 3A, after treatment with 50 μM cisplatin for 24 h, more colonies were observed in OV-90 and SK-OV-3 cell lines than that in IGROV-1 cells in the following several days.

Next, si-CHTOP was employed to investigate the role of CHTOP in epithelial ovarian cancer cisplatin-resistance. As shown in Figure 3B,C, compared with the mono-treatment of cisplatin, the combination treatment of CHTOP knockdown and cisplatin significantly weakened the colony formation ability of OV-90 and SK-OV-3 cells. Furthermore, the results from flow cytometry analysis showed that the early apoptosis fraction of both OV-90 and SK-OV-3 cells was markedly elevated by the combination treatment of CHTOP knockdown and cisplatin as compared with cisplatin mono-treatment (Figure 3D,E). Consistently, results from immunoblot analysis showed that, compared with cisplatin mono-treatment, several apoptosis-associated proteins, such as cleaved poly (ADP-ribose) polymerase 1 (PARP-1), active caspase-3, and Bcl-2-associated X protein (Bax), were significantly induced by the combination treatment of CHTOP silence and cisplatin in both OV-90 and SK-OV-3 cell lines, whereas anti-apoptosis-associated proteins, including B-cell lymphoma 2 (Bcl-2) and B-cell lymphoma-extra large (Bcl-xL), were remarkably inhibited by the combination of CHTOP silence and cisplatin (Figure 3F,G). In addition, results from TUNEL assay indicated that the combination of CHTOP silence and cisplatin caused much more DNA breaks than cisplatin mono-treatment in both OV-90 and SK-OV-3 cells as evidenced by the increased percentage of TUNEL-positive cells in the combination group (Figure 3H,I). Consistently, the protein expression of a DNA double-strand break marker, γH2AX, was significantly induced by the combination of CHTOP silence and cisplatin (Figure 3H,I).

### CHTOP knockdown decreased the stemness of epithelial ovarian cancer cells

**Figure 4:**
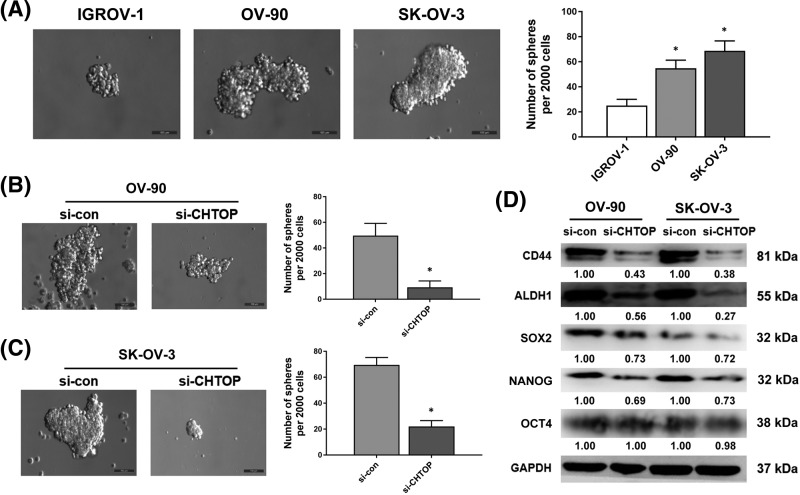
CHTOP knockdown decreased the stemness of epithelial ovarian cancer cells (**A**) OV-90 and SK-OV-3 cell lines showed an enhanced stemness as compared with IGROV-1 cell line. Cell line stemness was determined by the mammosphere-formation assay. The number of spheres was counted and compared after 5 days. Representative images were obtained at 200× magnification. (**B,C**) CHTOP knockdown significantly reduced the mammosphere-formation efficiency of OV-90 and SK-OV-3 cell lines. Cell line stemness was determined by the mammosphere formation assay. The number of spheres was counted and compared after 5 days. Representative images were obtained at 200× magnification. (**D**) CHTOP knockdown significantly decreased the protein expressions of several cancer stem cell markers. The protein expressions of representative cancer stem cell markers were tested and quantitated by immunoblot analysis and densitometric scan, respectively. GAPDH was used as the loading control. Data were expressed as mean ± S.E.M. (*n*=3). *P<0.05 vs IGROV-1 or si-con group.

As stemness has been considered as a critical mechanism underlying cancer metastasis and relapse, we, thus, also investigated the role of CHTOP in epithelial ovarian cancer stemness. To start this, the mammosphere-formatting ability of IGROV-1, OV-90, and SK-OV-3 cell lines was first compared. The results from this assay showed that, compared with IGROV-1, OV-90, and SK-OV-3 cell lines were characterized by a higher mammosphere formation efficiency (Figure 4A). More importantly, CHTOP silence can significantly reduce the number of spheres in both OV-90 and SK-OV-3 cells after 5 days (Figure 4B,C).

To further uncover the association of CHTOP with epithelial ovarian cancer stemness, the protein expressions of several representative cancer stem cell markers were also tested by western blot analysis. The results showed that, compared with the corresponding si-con group, the protein expressions of CD44, aldehyde dehydrogenase 1 (ALDH1), SRY (sex determining region Y)-box 2 (SOX-2), and NANOG in OV-90 and SK-OV-3 cells were significantly decreased by CHTOP knockdown (Figure 4D). However, CHTOP knockdown exerted no effect on the protein expression of octamer-binding transcription factor 4 (OCT-4) in both OV-90 and SK-OV-3 cells.

## Discussion

**Figure 5:**
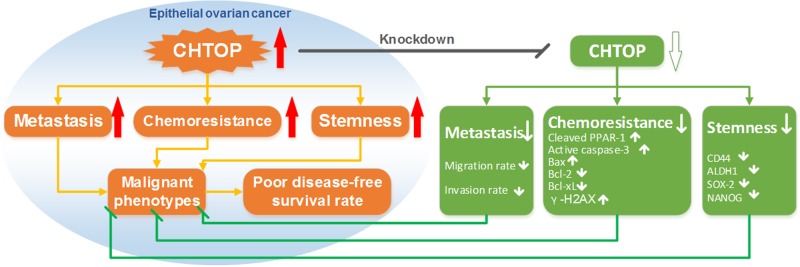
The proposed role of CHTOP in the malignant phenotypes of epithelial ovarian cancer

In the present study, we demonstrated the associations of CHTOP with the malignant behaviors of epithelial ovarian cancer cells. We found that CHTOP silence abolished the phenotypes of metastasis, chemoresistance, and stemness in epithelial ovarian cancer cells as summarized in Figure 5. These findings contribute to the understanding of the mechanism underlying epithelial ovarian cancer malignant phenotypes and provide a potential marker for both screening novel therapeutic agents and monitoring the clinical response to conventional therapy in ovarian cancer treatment.

The higher expression of CHTOP was specifically observed in epithelial ovarian cancer cells, which makes it possible to use CHTOP-targeted therapy in epithelial ovarian cancer with normal cells largely unaffected. This result was further confirmed in the comparisons between normal/adjacent tissues and tumor tissues from human beings. More importantly, the higher expression of CHTOP in ovarian cancer tissues was closely associated with a lower DFS rate, implying that CHTOP overexpression may exert important effect on epithelial ovarian cancer progress, which underlies the rationale of investigating the role of CHTOP in epithelial ovarian cancer.

Metastasis is one of the major causes of ovarian cancer therapeutic failure. Ovarian cancer cells in peritoneal cavity can invade the lined surface and grow above the peritoneal reflection in the pelvis [11]. Although surgery usually involves an *en bloc* resection of the ovarian tumors, reproductive organs, the sigmoid colon, and a primary bowel reanastomosis, micrometastasis of epithelial ovarian cancer cells via epithelial–mesenchymal–epithelial transition (EMT) did exist and accounted for many recurrence and death cases [12,13]. Therefore, in our study, we examined the role of CHTOP in epithelial ovarian cancer metastasis. Our results indicated that, compared with IGROV-1 cells, higher expression of CHTOP was closely correlated with a higher migration and invasion potential in SK-OV-3 and OV-90 cells, while CHTOP knockdown can significantly decrease their metastatic ability, suggesting that CHTOP has an essential role in epithelial ovarian cancer metastasis.

Chemotherapy is a major therapeutic option for ovarian cancer patients either in systematic or adjuvant situations. In this case, patients often receive chemotherapy with platinum (usually cisplatin or carboplatin) and a taxane (paclitaxel or docetaxel) [14]. It was reported that intraperitoneal chemotherapy can increase DFS time by 5 months and OS time by 15 months when compared with intravenous therapy [15]. However, chemotherapy may become ineffective after several cycles of therapy. The mechanisms underlying this include failure of intracellular drug accumulation, overactivation of antioxidant signaling, increase in DNA repair efficiency, and overactivation of anti-apoptotic signaling [16–18]. In this study, we found that higher expression of CHTOP in SK-OV-3 and OV-90 cells was associated with an enhanced cisplatin-resistant phenotype through chemosensitivity assay. In contrast, compared with the mono-treatment of cisplatin, CHTOP knockdown along with cisplatin not only reduced the colony-formatting ability of SK-OV-3 and OV-90 cells but also increased the cell apoptosis in both early (results from flow cytometry) and late stage (DNA breaks by TUNEL assay). Furthermore, results from immunoblot revealed that CHTOP inhibition further enhanced cisplatin-induced activation of apoptotic signaling (cleaved PPAR-1, active caspase-3, and Bax) and suppressed the activation of anti-apoptotic signaling (Bcl-2 and Bcl-xL). Of note, we observed that single CHTOP knockdown can significantly inhibited the colony formation ability of OV-90 and SK-OV-3, while apoptosis analysis showed that single CHTOP silence can induce early apoptosis, which is also evidenced by western blot analysis. Thus, these results suggest that CHTOP knockdown can trigger pro-apoptotic initiation of ovarian cancer cells which might be essential for cisplatin sensitization and CHTOP up-regulation may be associated with the development of resistance to chemotherapy-induced apoptosis in epithelial ovarian cancer.

Cancer stem cell has gained importance in explaining the aggressiveness of cancer. These special cells are characterized by a self-renewal ability and the capacity for replicating the heterogeneity of a whole tumor [19,20]. More importantly, they also show the strong resistance to conventional therapy and tumor-initiating potential, though constituting less percent of the tumor mass, which leads to cancer recurrence or metastasis [21]. Targeting these cells thus represents a perspective way to combat intractable cancer. Takai et al. [9] found that CHTOP can promote the tumorigenicity of glioblastoma cells. This action was mediated by the 5hmC that recruited the CHTOP–methylosome complex to methylate H4R3 and activated the transcription of several oncogenic genes [9]. Hypothesizing that CHTOP may play an important role in the tumorigenic ability of epithelial ovarian cancer cells, we also investigated the association of CHTOP with epithelial ovarian cancer stemness. The mammosphere formation assay is a golden-standard method for testing cancer stemness, which best mimics the tumorigenic process of cancer stem cells. Therefore, in this study, mammosphere culture method was employed to evaluate the stemness of epithelial ovarian cancer cells. In our results, we observed that SK-OV-3 and OV-90 had stronger stemness when compared with IGROV-1. In contrast, CHTOP silence significantly decreased the number of spheres in those two cell lines as compared with the corresponding si-con group, indicating that CHTOP overexpression is closely associated with an enhanced stemness in epithelial ovarian cancer. Further results from immunoblot suggest that CHTOP knockdown not only decreased the protein expressions of two representative surface markers of cancer stem cell (CD44 and ALDH1) but also inhibited the protein expressions of two important transcriptional makers of cancer stem cell (SOX-2 and NANOG). These results indicate that CHTOP inhibition can decrease the stemness of epithelial ovarian cancer cells by inhibiting the stemness-related signaling.

In summary, our results reveal that the overexpression of CHTOP is associated with an enhanced phenotype of metastasis, chemoresistance, and stemness in epithelial ovarian cancer cells, while CHTOP inhibition can abolish the malignant behaviors of epithelial ovarian cancer cells, which makes it possible to improve current ovarian cancer treatment by targeting CHTOP. However, the mechanisms underlying CHTOP in regulating cancer metastasis, apoptosis, and stemness remain unclear and no further studies can be referenced. Therefore, further investigation is required to uncover how CHTOP regulates the central genes related to metastasis, apoptosis, and stemness. For example, if there is any association between CHTOP and EMT and whether CHTOP regulates key genes involved in EMT (E-cadherin and N-cadherin), apoptosis-related signaling (Bcl-2, Bax, and Bcl-xL), and stemness signaling (SOX-2 and NANOG) at transcriptional or mRNA level. In addition, animal study is desired to further assess the therapeutic value of CHTOP inhibition in epithelial ovarian cancer. Findings from these studies will provide solid evidence for evaluating whether CHTOP can be used as an indicator for ovarian cancer prognosis or a therapeutic target for ovarian cancer treatment.

## Supporting information

**Supplementary Figure S1:** CHTOP knockdown inhibited the cell proliferation rates of OV-90 and SKOV-3 cells. OV-90 and SK-OV-3 cells were treated with si-con and si-CHTOP for 72 h and then the proliferation rates of OV-90 and SK-OV-3 cells were detected using a commercial kit during 7 consecutive days. The proliferation rates of OV-90 and SK-OV-3 cells in si-CHTOP group were obviously decreased compared with the corresponding si-con group. Data were expressed as mean ± S.E.M. (n=5).

**Supplementary Figure:** 

## Abbreviations

ALDH1: aldehyde dehydrogenase 1
Bcl-xL: B-cell lymphoma-extra large
Bcl-2: B-cell lymphoma 2
bFGF: basic fibroblast growth factor
CHTOP: chromatin target of protein arginine methyltransferase
DFS: disease-free survival
EGF: epithermal growth factor
EMT: epithelial–mesenchymal transition
ERα: estrogen receptor α
FBS: fetal bovine serum
OCT-4: octamer-binding transcription factor 4
OS: overall survival
PARP-1: poly (ADP-ribose) polymerase 1
PRMT: protein arginine methyltransferase
RIPA: radioimmunoprecipitation assay buffer
si-CHTOP: CHTOP-specific siRNA
SOX-2: SRY (sex determining region Y)-box 2
5hmC: 5-hydroxymethylcytosine
