# Supplementary material for: Chromatin target of protein arginine methyltransferase regulates invasion, chemoresistance, and stemness in epithelial ovarian cancer
Source: Biosci Rep. 2019 Apr 16;39(4):BSR20190016. doi: 10.1042/BSR20190016 (PMC6465198; doi:10.1042/BSR20190016)
Supplement: Supplementary file 1 [file bsr20190016_Supp1.pdf]

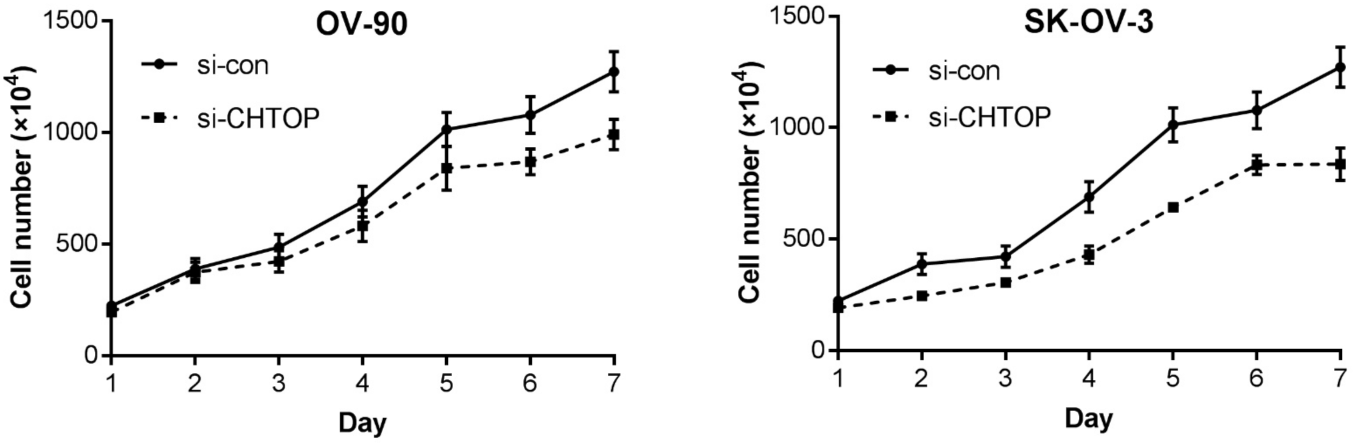

Supplementary Figure S1. CHTOP knockdown inhibited the cell proliferation rates of OV-90 and SK-OV-3 cells.

OV-90 and SK-OV-3 cells were treated with si-con and si-CHTOP for 72 h and then the proliferation rates of OV-90 and SK-OV-3 cells were detected using a commercial kit during 7 consecutive days. The proliferation rates of OV-90 and SK-OV-3 cells in si-CHTOP group were obviously decreased compared with the corresponding si-con group. Data were expressed as mean ± S.E.M. (n=5).

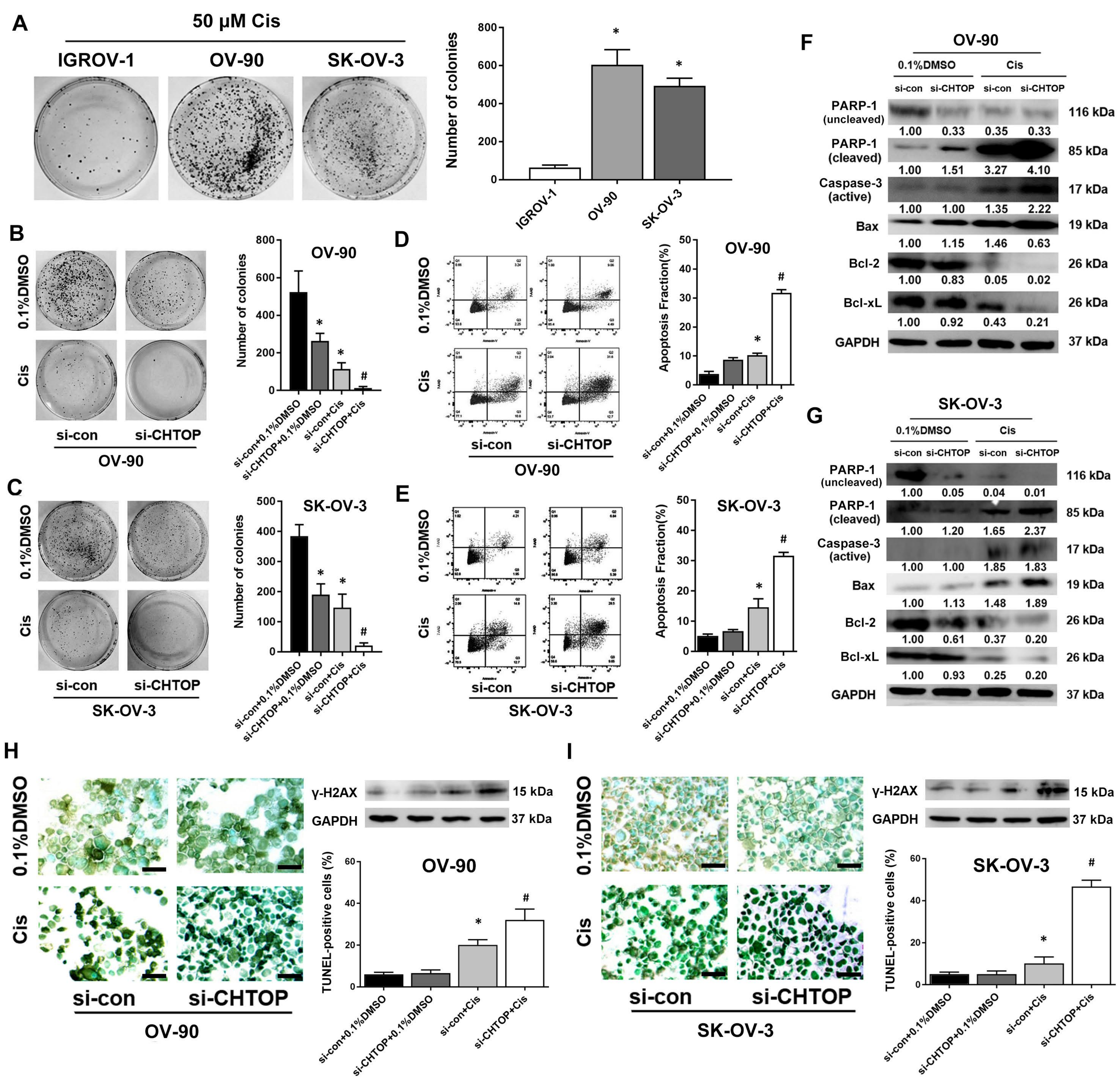
A
50 μM Cis
IGROV-1
OV-90
SK-OV-3
Number of colonies
B
0.1%DMSO
Cis
si-con
si-CHTOP
OV-90
Number of colonies
si-con+0.1%DMSO
si-CHTOP+0.1%DMSO
si-con+Cis
si-CHTOP+Cis
C
SK-OV-3
D
Apoptosis Fraction(%)
E
F
PARP-1 (uncleaved)
1.00 0.33 0.35 0.33
116 kDa
PARP-1 (cleaved)
1.00 1.51 3.27 4.10
85 kDa
Caspase-3 (active)
1.00 1.00 1.35 2.22
17 kDa
Bax
1.00 1.15 1.46 0.63
19 kDa
Bcl-2
1.00 0.83 0.05 0.02
26 kDa
Bcl-xL
1.00 0.92 0.43 0.21
26 kDa
GAPDH
37 kDa
G
PARP-1 (uncleaved)
1.00 0.05 0.04 0.01
116 kDa
PARP-1 (cleaved)
1.00 1.20 1.65 2.37
85 kDa
Caspase-3 (active)
1.00 1.00 1.85 1.83
17 kDa
Bax
1.00 1.13 1.48 1.89
19 kDa
Bcl-2
1.00 0.61 0.37 0.20
26 kDa
Bcl-xL
1.00 0.93 0.25 0.20
26 kDa
GAPDH
37 kDa
H
γ-H2AX
15 kDa
GAPDH
37 kDa
TUNEL-positive cells (%)
I
